# The deuterium/hydrogen distribution in chondritic organic matter attests to early ionizing irradiation

**DOI:** 10.1038/ncomms9567

**Published:** 2015-10-13

**Authors:** Boris Laurent, Mathieu Roskosz, Laurent Remusat, François Robert, Hugues Leroux, Hervé Vezin, Christophe Depecker, Nicolas Nuns, Jean-Marc Lefebvre

**Affiliations:** 1 UMET, Université Lille 1, CNRS UMR 8207, Villeneuve d'Ascq F-59655, France; 2 IMPMC, CNRS UMR 7590, Sorbonne Universités, Université Pierre et Marie Curie, IRD, Muséum National d'Histoire Naturelle, CP 52, 57 rue Cuvier, Paris 75231, France; 3 LASIR, Université de Lille 1, CNRS UMR 8516, Villeneuve d'Ascq F-59655, France; 4 Institut M.E. Chevreul, Université de Lille 1, CNRS, FR 2638, Villeneuve d'Ascq F-59655, France

## Abstract

Primitive carbonaceous chondrites contain a large array of organic compounds dominated by insoluble organic matter (IOM). A striking feature of this IOM is the systematic enrichment in deuterium compared with the solar hydrogen reservoir. This enrichment has been taken as a sign of low-temperature ion-molecule or gas-grain reactions. However, the extent to which Solar System processes, especially ionizing radiation, can affect D/H ratios is largely unknown. Here, we report the effects of electron irradiation on the hydrogen isotopic composition of organic precursors containing different functional groups. From an initial terrestrial composition, overall D-enrichments and differential intramolecular fractionations comparable with those measured in the Orgueil meteorite were induced. Therefore, ionizing radiation can quantitatively explain the deuteration of organics in some carbonaceous chondrites. For these meteorites, the precursors of the IOM may have had the same isotopic composition as the main water reservoirs of the inner Solar System.

## Abstract (web summary)

The insoluble organic matter in primitive carbonaceous chondrites has a systematic large enrichment in deuterium and several hypotheses have been proposed to explain this. Here, the authors demonstrate that irradiation from the protosun could quantitatively explain the deuteration.

## Graphical abstract

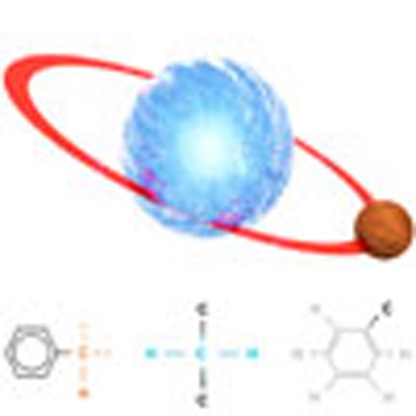

Carbonaceous chondrites result from the accretion of materials formed during the early stages of the Solar System's history. These meteorites typically contain a large amount (up to 4 wt%) of organic matter, which was formed by abiotic processes^1^ and occurs as small grains in the fine-grained matrix^2^ (from a few hundred nanometres to a few micrometres in diameter). The insoluble organic matter (IOM) consists of small aromatic units, which are highly substituted and are connected by short and branched aliphatic chains^3^,^4^. The presence of monoradicals and diradicals is a major structural feature of extraterrestrial IOM, and they are rarely found in terrestrial counterparts^5^. Another striking feature of this IOM is the systematically large enrichment in deuterium compared with the solar hydrogen reservoir and the Jovian atmosphere^6^,^7^. The bulk hydrogen isotopic composition of the IOM also contrasts significantly with terrestrial signatures (δ*D*=972±2‰ for the (Ivuna-like) carbonaceous chondrite Orgueil and up to 3,527‰ for the (Renazzo-like) carbonaceous chondrite LEW85332, relative to standard mean ocean water (SMOW)^8^). In addition, the δ*D* of the IOM of Orgueil is heterogeneous at the molecular scale^9^: the isotopic ratio depends on the hydrogen position in the macromolecule (for example, the type of C–H bond). Several mechanisms, occurring either in the interstellar medium^10^,^11^,^12^, the protosolar nebula^9^,^13^ or later on in parent asteroids^14^, have been proposed to explain the isotopic signature of the IOM. These mechanisms include ion-molecule reactions at low temperatures in molecular clouds^15^,^16^, water reduction and subsequent isotopic exchange with organic molecules during aqueous alteration^14^, or a synthesis from ice grains catalysed by the ultraviolet irradiation^17^.

In the protosolar nebula, charged particles (mainly protons and electrons) forming the solar wind are streamed along magnetic lines through the protoplanetary disk, and they constantly collide with micrometre-sized dust particles. The interactions of these charged particles with matter are dominated by an ionizing mechanism similar to the effects of energetic photons (ultraviolet and X-rays). Ionizing irradiation refers to an interaction with the electron shells of the target atoms. After such interaction, the electronic structure of the target is at least temporarily affected. The main consequences for the target are ionization and breaking of chemical bonds, but they do not include direct ejection of atoms from the target. During the T-Tauri stage of the Sun's evolution, such irradiation was very efficient because the solar flux was larger than at present^18^ by three orders of magnitude^19^. Protons may interact with the nuclei of atoms, causing structural modification and sputtering, but these effects are restricted to the surfaces of solids; the penetration depth of protons is limited to a few tens of nanometres for an energy typical of solar wind particles (for example, within the range of 1–10 keV). For electrons, damage results mainly from the interactions with the electron shells of the target atoms, and the contribution of nuclear interactions (such as sputtering) is negligible. However, electrons induce ionization at depth that can cause chemical, structural and isotopic modifications. Recently, significant D-enhancements were produced by irradiating organic macromolecules with the electron beams generated by transmission and scanning electron microscopes (SEM)^20^,^21^,^22^. Such ionizing irradiation was also shown to produce monoradicals and diradicals^22^. However, these studies were unable to derive intramolecular fractionation factors (*α*) in relation to the different types of C–H bonds present in the IOM and their possible links with the *α* values of the chondritic IOM^5^. Consequently, no direct comparison could be made between the experimental results and the D/H signatures measured in the IOM.

Here, we present the effects of electron irradiation on the D/H fractionation of organic precursors containing different functional groups to derive typical intramolecular fractionation factors. Electrons were selected as ionizing radiation because their ionizing efficiency and their penetration depth are both higher than other candidates (ultraviolet photons and protons). Consequently, electron irradiations are easier to carry out in the laboratory, irradiated materials are easier to characterize by conventional spectroscopic and isotopic methods, and derived properties are bulk properties rather than surface properties. For comparison, we have also performed some ultraviolet irradiation experiments (see Supplementary Note 1 for details). The results suggest that the nature of the incoming radiation may not have a determining effect.

## Results

### Experimental strategy

Despite being structurally distinct from the IOM, polymers are suitable analogues. Polymers and IOM share the same functional groups (for example, aliphatic chains and aromatic cycles). Yet, polymers consist of long chains composed of many repeated units (monomers), whereas the IOM does not show any significant repetition of any pattern. Three polymers, polyethylene terephthalate (PET, (C_10_H_8_O_4_)_*n*_), polystyrene (PS, (C_8_H_8_)_*n*_) and polyethylene (PE, (C_2_H_4_)_*n*_)), were chosen to derive the intramolecular fractionation factors between the moieties identified in the IOM. Each polymer exhibits specific and distinct combinations of different types of C–H bonds. In PS, five out of eight of H atoms are bonded to aromatic carbon atoms (hereafter referred to as ‘ring'), two out of eight to aliphatic carbon (CH_2_) and one out of eight to benzylic carbon (CH). Linear aliphatic chains of CH_2_ characterize PE, whereas half of the C–H bonds are aliphatic and half are aromatic in PET. The bulk initial hydrogen isotope compositions of these three polymers, assumed to be homogeneous, were close to SMOW and were typical of manufactured organic products (PET: −33±8‰, PS: −30±10‰ and PE: −76±7‰).

Thin films (ranging from 900 nm up to 10 μm) were irradiated in a SEM at 30 keV and room temperature. The electron doses (that is, the amount of deposited energy per unit volume) ranged from 1.6 × 10^24^ to 1.4 × 10^25^ eV cm^−3^. These doses correspond to typical exposure durations as short as a few hundred years, according to the current understanding of the dynamics of protoplanetary disks^18^,^19^,^23^ and taking into account the fact that direct ionizing irradiation is essentially effective on a thin layer of the disk surface (see the Methods section). Samples were then characterized by using Fourier–Transform Infrared Spectroscopy (FTIR), Time of Flight Secondary Ion Mass Spectrometry (ToF-SIMS) and nano Secondary Ion Mass Spectrometry (NanoSIMS; see the Methods section).

### Effects of electron irradiation

**Figure 1:**
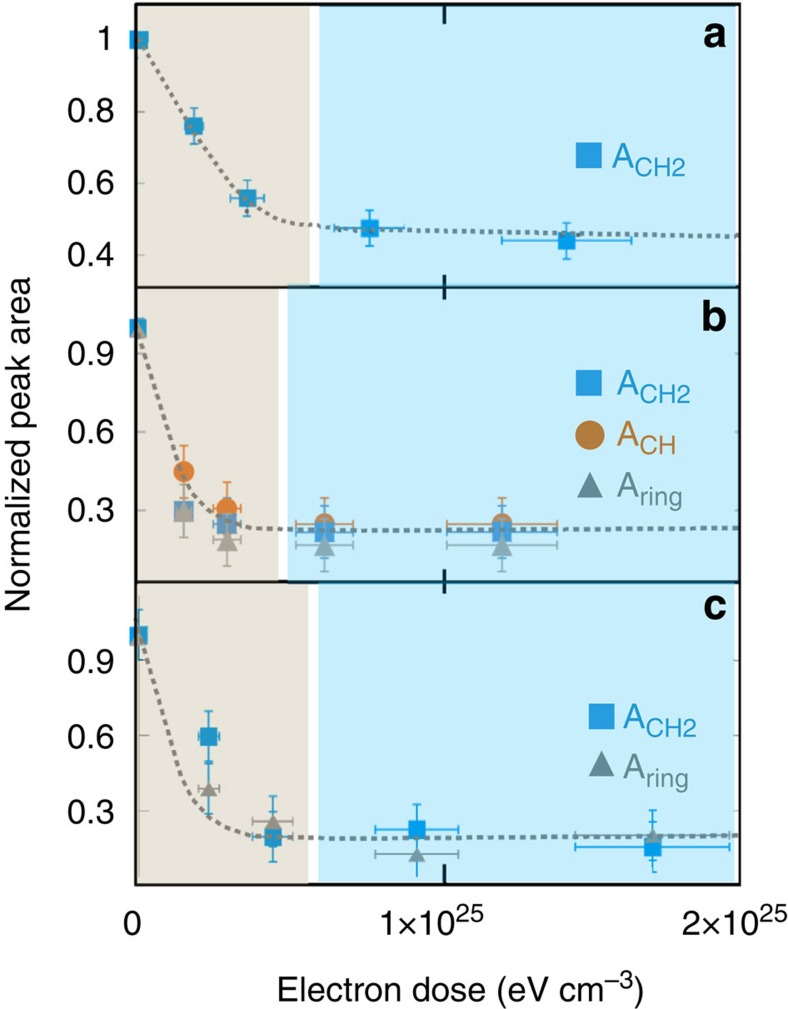
Evolution of the infrared peak area of C–H functional groups. All of the experiments were performed at 30 keV and 300 K. This evolution is documented for (**a**) polyethylene (PE), (**b**) polystyrene (PS) and (**c**) polyethylene terephthalate (PET). For each sample, the intensity of the infrared signal related to CH_2_ (1,465 cm^−1^), CH (2,890 cm^−1^) and ring (3,030 cm^−1^) are considered. The peak areas (namely *A*_CH2_, *A*_CH_, *A*_ring_) are normalized to the peak area of non-irradiated starting materials. On the three panels, the blue region represents the plateau, whereas structural, compositional and isotopic changes are still functions of the electron dose in the grey area. The doses required to reach 90% of the structural plateau delimit the blue and grey regions of the plots.

**Figure 2:**
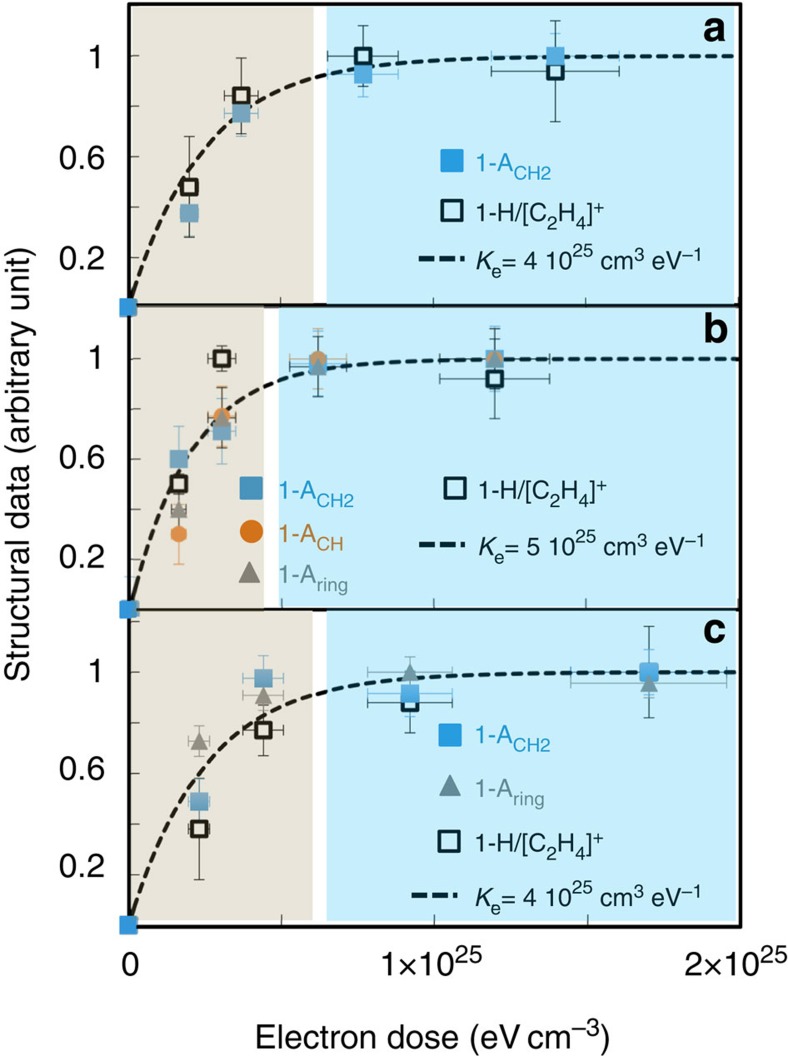
Structural and compositional evolution of the three polymers. All of the experiments were performed at 30 keV and 300 K. A combination of ToF-SIMS (H^+^/C_2_H_4_^+^) and FTIR data (the peak areas *A*_CH2_, *A*_CH_, *A*_ring_) are presented for (**a**) polyethylene (PE), (**b**) polystyrene (PS) and (**c**) polyethylene terephthalate (PET). Data are all normalized between 0 and 1, allowing a direct comparison of different physical quantities. All evolutions are relative to the properties of the non-irradiated precursor. The coefficient *K*_E_ is the electron dose constant obtained from a first-order rate equation (see the Methods section for details). On the three panels, the blue region represents the plateau, whereas structural, compositional and isotopic changes are still functions of the electron dose in the grey area. The doses required to reach 90% of the structural and chemical plateaus delimit the blue and grey regions of the plots. Data for PET are from ref. 22.

Electron irradiation of the three polymers affects their structures in a similar way. First, for each polymer, the intensity of the infrared signal decreases relative to all of the C–H groups, namely CH_2_ (1,465 cm^−1^), CH (2,890 cm^−1^) and ring (3,030 cm^−1^), at similar rates (Fig. 1). This indicates that all C–H bonds are simultaneously affected by irradiation. Hence, the decrease of the signal of the functional C–H groups is independent of the dissociation energy of individual C–H bonds. Second, these infrared features and the decrease of the H^+^/C_2_H_4_^+^, measured by ToF-SIMS, follow a comparable trend for the three polymers (Fig. 2). All of these structural and compositional data can be described by a first-order rate equation. The dose constant (*K*_E_) derived from this formalism describes the rate dependence of the structural evolution for each analogue versus energy deposition. For the three polymers, the *K*_E_ values are comparable (4 × 10^−25^ cm^3^ eV^−1^ for PE and PET, 5 × 10^−25^ cm^3^ eV^−1^ for PS, also see the Methods section). This indicates that both C–H bond break-up and hydrogen loss from the polymer films occurred at comparable rates for all polymers.

**Figure 3:**
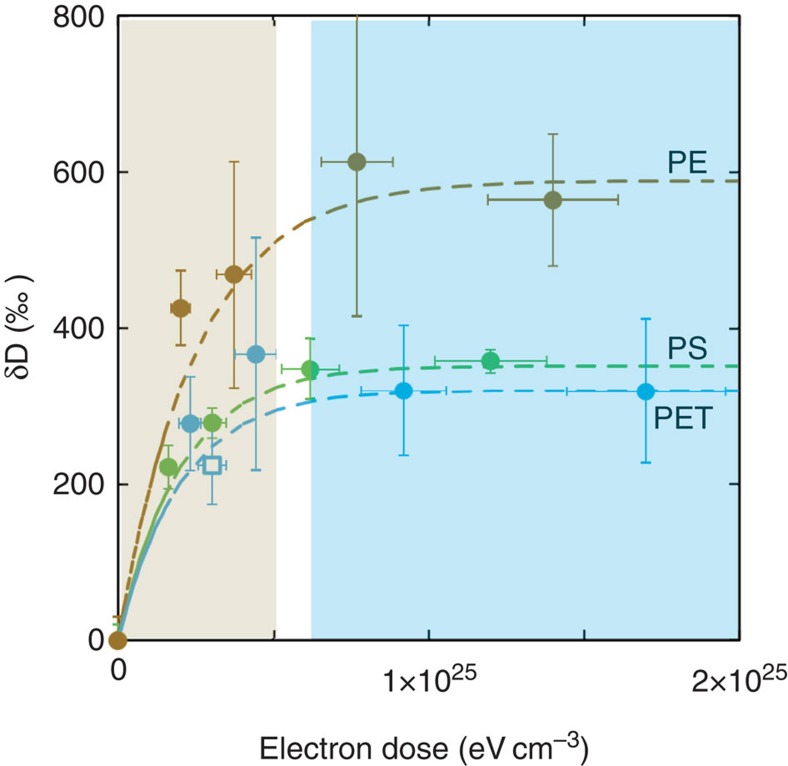
Isotopic evolution of the three polymers. Isotopic compositions (δ*D*) for polyethylene (PE—brown symbols), polystyrene (PS—green symbols) and polyethylene terephthalate (PET—blue symbols) are presented relative to the non-irradiated starting materials. All of the experiments were performed at 30 keV and 300 K. Dashed lines represent the calculated trends given by the first-order rate equations. The same *K*_E_ values were used for isotopic and structural evolutions. The blue region represents the plateaus, whereas structural, compositional and isotopic changes are still functions of the electron dose in the grey area. The doses required to reach 90% of the isotopic plateaus delimit the blue and grey regions of the plots (these doses are different for each analogue but sit within the white intermediate region of the plot). An additional datum (blue open square), corresponding to the isotopic signature of a PET film irradiated by ultraviolet, is also provided. It plots along with other data collected on PET films irradiated by electrons. The calculations of both electron and ultraviolet doses are detailed in the Methods section.

More importantly, the strong correlation between the structural, compositional and isotopic evolutions previously established for the PET film^22^ is confirmed for all samples. The aforementioned first-order rate equation and *K*_E_ dose constant derived from structural and compositional data were used to describe the isotopic data set. No additional adjustments were required to produce remarkable fits to the data (Fig. 3). As the electron dose increases, the δ*D* of each polymer increases until it reaches a plateau value (see the Methods section). Here, the major feature is that each polymer exhibits a different isotopic plateau, despite their comparable rates of structural and compositional evolution. These plateau values correspond to the maximum bulk isotopic compositions (relative to the non-irradiated starting materials), with δ*D* values of 463±113‰, 315±30‰ and 272±60‰ for PE, PS and PET, respectively. This is a consequence of the nature and the relative proportions of the different C–H groups present in the starting material.

## Discussion

The three studied polymers have different D/H plateau values. The nature and the relative proportions of the different C–H groups therefore control the extent of the bulk isotopic signature under ionizing irradiation. From a plateau value, the individual isotopic signatures of aliphatic, aromatic and benzylic C–H bonds of the irradiated polymers can be calculated by mass balance, taking into account the relative proportions of these groups in the three different polymers. A single set of values is determined thanks to three independent mass balance equations (see the Methods section): δ*D* (vs SMOW) values of 81±128‰, 463±113‰ and 1,192±173‰ for aromatic, aliphatic and benzylic C–H groups, respectively. Assuming local equilibrium, the intra-molecular fractionation factors (for example, *α*_A-B_≈(δ*D*_A_+1,000)/(δ*D*_B_+1,000)) associated with the irradiation process were derived: *α*_benzylic-aromatic_=2.03±0.29 and *α*_aliphatic-aromatic_=1.35±0.19. These values are in remarkable agreement with the intramolecular fractionation factors determined from the IOM of the Orgueil meteorite (Table 1), for which the measured fractionation factors are *α*_benzylic-aromatic_=1.96±0.05 and *α*_aliphatic-aromatic_=1.35±0.05^9^. Furthermore, our results are also in agreement with the fractionation factors characterizing the isotopic equilibrium between organic molecules and a deuterium plasma (D_3_^+^)^24^. This match may suggest that the ionizing irradiation induces a local isotopic equilibration between the irradiated solid and the hydrogen-rich plasma. This assertion is further sustained by the lack of significant isotopic exchange between organic insoluble macromolecules and H_2_, H_2_O or other neutral volatile species under ambient conditions^24^. Our study shows that the heterogeneous distribution of H isotopes at the molecular scale in Orgueil IOM is likely a result of an ionizing irradiation process on different types of C–H groups.

Another excellent agreement is found between the δ*D* (relative to SMOW) derived for each C–H group of irradiated samples and the δ*D* measured in the IOM of Orgueil. Hence, if the isotopic signature of Orgueil was shaped by irradiation, the bulk isotopic composition of the precursor of the IOM before irradiation was very similar to the composition of our starting material, being very close to SMOW. The composition of the water trapped in Orgueil is also commensurate with SMOW^14^. Therefore, our result suggests that both the precursor of the IOM and the water in Orgueil may have had the same initial isotopic composition as most of the water of the inner bodies. More generally, this new fractionating process could explain the systematic D-enrichment measured in the IOM relative to the water present in all carbonaceous chondrites^14^. Such a contrasting behaviour between water and organic matter would be consistent with the poor efficiency of the disk ionization in enhancing the D/H of the solar water reservoirs^25^.

In the protoplanetary disk, other solar ionizing particles, such as energetic photons and protons, may also have contributed to shaping the structural and isotopic signature of the IOM. Preliminary results of the structural and isotopic evolutions of a PET film irradiated by ultraviolet radiation suggest that the nature of the incoming ionizing particle may not be the pivotal factor. For a given deposited energy, the D/H and the nature of the radicals formed are comparable for ultraviolet and electron irradiation (Fig. 3, Methods section and Supplementary Fig. 4). However, each particle has its own characteristic penetration depth and ionizing efficiency. In this respect, electrons appear to be relatively versatile and are able to modify the target over several micrometres in depth. Still, our results call for a detailed comparison of the effects of these different forms of ionizing radiation at the molecular scale. Such a comparison could help to better understand the molecular processes responsible for the deuteration of organic precursors. These mechanisms are not yet clearly identified and may either operate during the interaction with the incoming particles or during the rearrangement/recombination of the material. The fact that, for a given deposited energy, ultraviolet and electron irradiation induce comparable D-enrichments suggests that fractionation may be primarily produced during recombination rather than during the interactions between a given type of particle and the organic precursors. However, this assertion must be experimentally confirmed because it would lead, in turn, to a more comprehensive description of the D/H evolution in organic matter in the protoplanetary disk regions exposed to ionizing radiation from the protosun. Finally, a better understanding of the irradiation-driven fractionation of other light elements (such as nitrogen, carbon and oxygen) should also shed new light on the nature and evolution of the extraterrestrial IOM and water reservoirs, and it might even extend our findings to CR chondrites that show strong enrichments in both heavy nitrogen and deuterium.

## Methods

### Sample preparation and irradiation

The PS samples were prepared by spin-coating deposition on a silicon wafer. Spin coating is a centrifugal force-driven method for the deposition of thin films on a flat substrate. The deposition was achieved by the rotation of the substrate, on which a small quantity of the solubilized film is spread. The PS was solubilized in toluene. For a speed of 2,000 r.p.m. and a concentration of 10 wt% PS in toluene, a final thickness of 1.3 μm was obtained. After the deposition, samples were stored under secondary vacuum conditions for several hours to ensure complete evaporation of toluene and polymerization of the film. The PE samples were derived from a 10-μm-thick film (GoodFellow Cambridge Limited) and were mounted to a brass sample holder using conductive copper tape. The PET consisted of a 900-nm-thick film that was biaxially oriented and semi-crystalline (from Good Fellow Co.), and it was deposited on a brass sample holder.

Electron irradiation experiments were carried out on a Hitachi S4700 field-emission gun scanning electron microscope at 30 keV (at the Electron Microscope Facility, University Lille 1). Electron fluences were measured with a Faraday cup. The onset of the melting of PE is typically lower than 80 °C. After irradiation, the film did not show any indication of thermal degradation. We can thus estimate the temperature of the samples under the electron beam to be between 20 and 80 °C. After irradiation experiments and between the different analytical steps, all samples were stored under a flow of dry argon in the dark.

Ultraviolet irradiations were performed on PET films. They were performed with a polychromatic ultraviolet lamp (Hamamatsu irradiation system) equipped with a band-pass filter at 239 nm. At this wavelength, the coupling between ultraviolet and the PET film was especially efficient^26^. The nominal energy of the lamp was 300 μW cm^−2^. However, the filter absorbed 80% of the incident energy at 239 nm so that the actual energy of the lamp during these experiments was estimated to be 60 μW cm^−2^.

### Determination of the energy deposited (electron and ultraviolet doses)

The modifications induced by electron irradiation are directly proportional to the energy deposited in the sample^22^. The fluence (that is, the number of electrons per surface unit) is an important parameter in the determination of the energy deposited by electrons in the target samples. Electron fluences were measured with a Faraday cup with a precision of ±15% (Supplementary Table 1). For irradiation performed at 30 keV, the energy is not entirely dissipated in the film, as the path followed by the electrons is longer than the thicknesses of the PE and PS samples (penetration depth up to 17.2 μm at 30 keV, see Supplementary Fig. 1). In addition, we calculated the energy deposition in the first few hundred nanometres, which is the typical thickness analysed by NanoSIMS for the D/H measurements. The energy deposited in the film directly depends on the electronic stopping power (that is, the linear rate of the energy transfer between the incoming particle and the target). Here, the characteristic electron path and the stopping power are taken from the ESTAR database^27^ (Supplementary Table 1). To take into account both the fluence and the stopping power, all of the isotopic and structural data are presented as a function of the *dose (E)*:


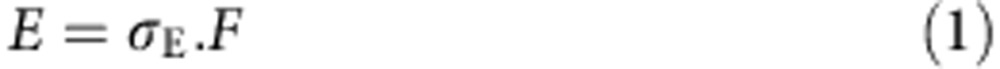


where *E* is the electron dose (eV cm^−3^), *σ*_E_ is the electronic stopping power (MeV cm^−1^) and *F* is the fluence (e^−^ cm^−2^).

A comparable approach was used for ultraviolet irradiation to directly compare the effects of ultraviolet and electron irradiation as a function of the energy deposited on the PET film. Here, an absorption coefficient of 2.5 × 10^5^ cm^−1^ was selected for photons with a wavelength of 239 nm (ref. 26). The calculated deposited energy after 26 h of irradiation is 3.1 × 10^25^ eV cm^−3^ within the first 100 nm of the PET films, which is the typical thickness analysed by NanoSIMS.

### Sample analysis

The evolution of the molecular structure of samples during irradiation was studied by infrared spectroscopy (FTIR) using the imager spotlight 300 from Perkin-Elmer at UMET, University Lille 1. Transmission spectra were collected by averaging 32 scans in the 750–4,000 cm^−1^ range. A linear baseline correction was applied to each spectrum between 750 and 3,700 cm^−1^. The peak areas were evaluated using Gaussian deconvolution (the commercial Peakfit software by Systat software, Inc. was used for this purpose). For the PS film, the infrared spectra were complicated by the presence of interference fringes. These fringes typically result from the thin sample (1.3 μm) synthetized by spin-coating deposition. The substrate and the film act as a plane–parallel interface from which interference patterns (sinusoidal waves) arise. These interferences (a sinusoidal contribution) were removed from the general spectra using a conventional subtraction method.

For all irradiated samples, a clear signal of adsorbed water is observed at the surface of the films (Supplementary Fig. 2). Furthermore, in the case of PE, the infrared spectra in the range 750–1,750 cm^−1^ present additional optical features that could not be attributed to any vibrational modes, based on the available literature. These features and the spectral contribution of adsorbed water make the analysis of structural changes very complicated and necessarily qualitative. For this reason, we only focused on the normalized evolution of the areas of the main characteristic peaks for each polymer but not on unattributed peaks or those originated from surface artefacts because of adsorbed water. In the case of the PE samples, the typical bands of adsorbed water were first subtracted from the spectra before quantification of the peak areas (Supplementary Fig. 2).

All of the D/H analyses were performed on the Cameca NanoSIMS 50 at MNHN Paris, France. The surfaces of the samples were gold-coated (20 nm thick) before analysis, and an electron-flooding gun was used during analysis. The sample surface was rastered by a primary Cs^+^ beam over areas of 8 × 8 μm^2^ divided into 64 × 64 pixels at a raster speed of 2 ms per pixel. The isotopes were collected from the inner 5.6 × 5.6 μm^2^ area in multicollection mode with a beam current of 16 pA. Secondary ions of H- and D- were collected by electron multipliers with a dead-time of 44 ns. The mass spectrometer was set to 4,000 mass resolving power. Before each analysis, a 400-pA primary beam was rastered over a 10 × 10 μm^2^ surface area over a period of 50 s to remove gold coating and surface contamination. This procedure was particularly important for removing the sample layers containing adsorbed water. For such polymers, this presputtering typically removes ∼50 nm of material before the analysis starts. The instrumental mass fractionation was corrected by measuring several times a day the unirradiated films used as internal standards. Turning to the instrumental drift, a daily measurement of a natural kerogen type III showed a reproducibility of ±30‰. A typical analysis consisted of bracketing measurements of irradiated areas by nearby unirradiated zones. Error bars reported in this study are 1*σ* errors based on the quadratic sums of standard deviations on replicates performed on non-irradiated (three to four analysis) and irradiated areas (two to four analysis). For each polymer and before irradiation, the δ*D* relative to the SMOW was determined by conventional gas source mass spectrometry (performed at CRPG, Nancy) and was found to be −33±8‰, −30±10‰ and −76±7‰ for PET, PS and PE, respectively.

The ToF-SIMS spectra measurements were carried out in positive mode using a ToF-SIMS V instrument (ION-TOF GmbH Germany) at UCCS, University Lille 1. This instrument is equipped with a Bi liquid metal ion gun. Pulsed Bi^+^ primary ions were used for the analysis (25 keV, 1 pA). Surface spectra were taken from an area of 500 × 500 μm^2^ (256 × 256 pixels—20 scans) and then reconstructed over 100 × 100 μm^2^ areas corresponding to irradiated and non-irradiated zones. Charging effects, due to the primary ion beam, were compensated for using pulsed low-energy electrons (20 eV). The C/H ratios for PS, PE and PET could not be directly determined from ToF-SIMS measurements. Instead, we quantitatively compared the intensity ratio H^+^/C_2_H_4_^+^ for each irradiated spot with the surrounding unirradiated material. This choice of using a molecular fragment instead of a simple ion (C^+^) is related to the analytical mode used for ToF-SIMS analysis. This setup has two advantages. First, it provides information on the evolution of the carbon skeleton through irradiation (from H^+^ to C_15_H_13_^+^ masses). After irradiation, the light masses (corresponding to short fragments of irradiated matter) are more abundant than heavier molecular fragments (Supplementary Fig. 3). This is the direct result of the chain scission caused by irradiation. Second, this setup reduces the carbon-based (C^+^) contamination during surface analysis. For these reasons, C_2_H_4_^+^ moieties were chosen as the proxy for documenting the change in the C/H ratio during irradiation. Only normalized quantities (relative to unirradiated samples) are provided here because the measured intensity ratios are not directly commensurable to the true atomic C/H ratio. Finally, as for isotopic measurements, a typical analysis consisted of bracketing measurements of irradiated spots and unirradiated zones. Error bars reported in this study are 1*σ* s.d.s of the replicates on non-irradiated areas (three to four analyses), and classical error propagation was applied to normalized values.

Concentrations of organic radicals were derived from electronic paramagnetic resonance (EPR). The EPR spectra were recorded with a Bruker Elexsys E500 operating at 9 GHz at LASIR, University Lille 1. Amplitude modulation and microwave power were set to 2 G and 5 mW, respectively. For ultraviolet irradiation, the film of PET (6 × 6 mm^2^ size) was directly irradiated inside the transmission mode high sensitivity (TMHS) cavity through optical windows. The spin concentration was determined using weak pitch with a double integration procedure. Typically, in this analytical configuration, the absolute error on the spin concentration is better than 5%.

### Kinetic analysis of structural and isotopic changes

All of the structural, chemical and isotopic changes presented in this study share a common kinetics during irradiation. A first-order rate law successfully describes such changes. Typically, a first-order rate law relates the time derivative of the concentration of a given species to its instantaneous concentration through a time constant. As the electron dose *E* is a function of both the stopping power and the electron fluence equation (1), the evolution of a quantity *A* is described as


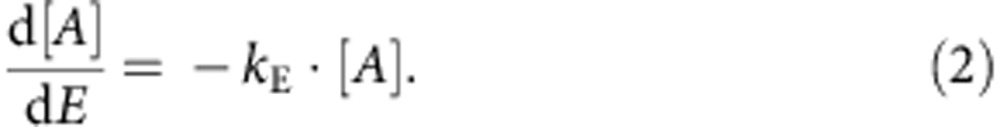


where *k*_E_ is the dose constant. The integration of this relation gives access to the evolution of the quantity *A* as a function of the electron dose:


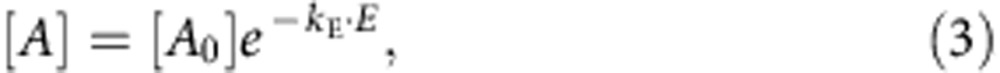


where [*A*_*0*_] is the initial intensity of a given feature of the polymer over an increasing dose *E*. The different patterns of change can be compared because the dose constant is a direct proxy for the rate of molecular change through irradiation. For this purpose, we used normalized quantities (peak areas and ratios).

Based on this fitting procedure, we determined the electron dose required for each sample to reach 90% of the plateau value (Supplementary Table 2). These doses were, in turn, used as threshold values to determine the isotopic signature of each sample at the plateau. All experiments performed at higher doses were taken into account to calculate the characteristic isotopic signature on the plateau. Typically, two data points for each polymer were found to sit on the plateau. The isotopic plateau values for each polymer were determined as the mean of the isotopic data situated on the plateau. The error was taken as the 1*σ* standard deviation of such data set.

### Determination of intramolecular fractionation factors

As for the structural evolution, the isotopic data set shows isotopic plateaus (Fig. 3). The isotopic signatures of irradiated materials at their respective plateaus (given relative to SMOW) are 463±113‰, 315±30‰ and 272±60‰ for PE, PS and PET, respectively. The isotopic signature of each C–H bond was then determined assuming local equilibrium under the electron beam. A simple mass balance was considered because the initial relative proportions of the C–H bonds are known, and all C–H groups are lost at a comparable rate during irradiation, as discussed above. Hence:


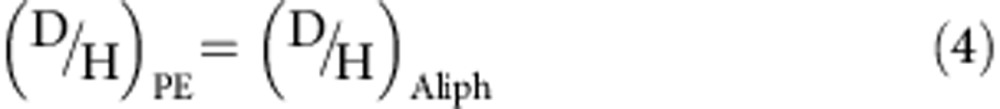



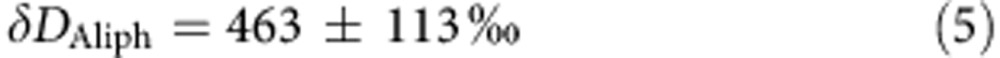



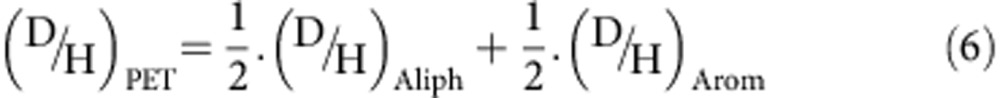



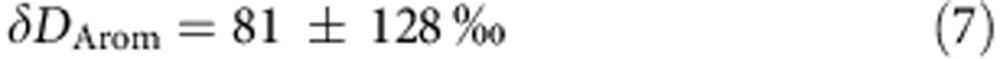



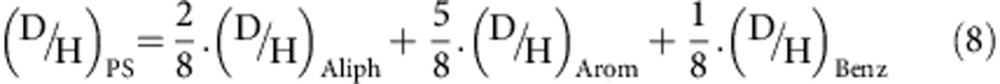



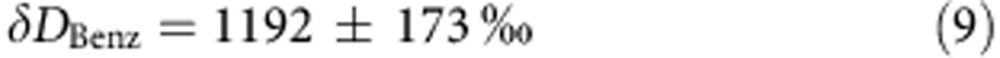


From these molecular δ*D* (relative to SMOW), the intramolecular fractionation factors were easily determined:


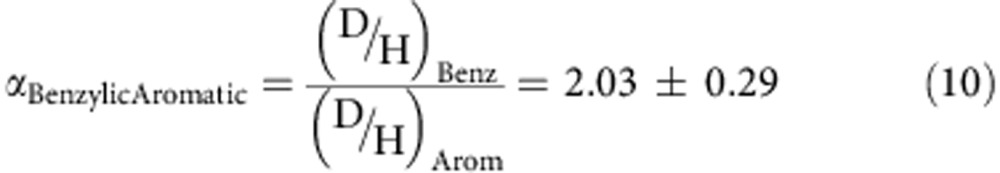



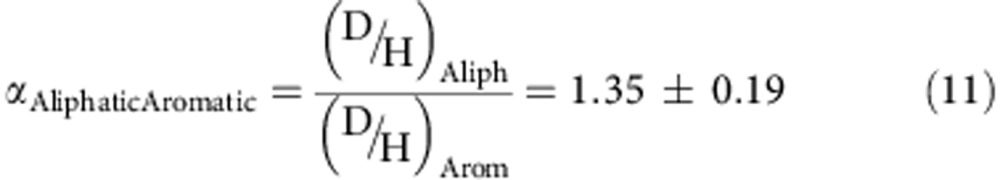


The errors of the ratios were propagated to the *α* values (quadratic sums), and the relative errors of the factors were found to be lower than 14%.

### Timescale for a similar irradiation in the disk

In a protoplanetary context, the timescale required for the irradiation to reach a dose comparable to those studied here was determined from the measured present-day energy distribution of the quiescent solar flux^18^. This distribution follows a power law where low-energy electrons (from 1 to 10 keV) are much more abundant than high-energy ones (Supplementary Fig. 5). For the low-energy range (1–10 keV), the integrated flux and the associated electron dose rate were calculated (blue and red areas in Supplementary Fig. 5). The stellar activity of the protostars has been shown to be at least three orders of magnitude higher than today^19^,^28^. Thus, we considered an electron flux higher than the quiescent present-day solar flux by three orders of magnitude. In this context, only few hundred years are needed to produce the same deposited energy that was experienced in our experimental irradiations (Supplementary Table 3).

Finally, protoplanetary environments are optically thick and cold. Therefore, direct electron (photon or ion) irradiation is essentially effective on a thin and warmer layer of the disk surface. Conversely, close to the midplane, the dust is not exposed to solar particles. This shield effect is, however, poorly efficient because of the vertical motions predicted to occur in a turbulent disk^23^. Current models show that micrometre-sized dust from the disk might have spent up to 5% of its lifetime at altitudes where irradiation is efficient^23^. Therefore, the doses required to reach the plateau values are completely compatible with the typical lifetime of the disk.

## Additional information

**How to cite this article:** Laurent, B. *et al*. The deuterium/hydrogen distribution in chondritic organic matter attests to early ionizing irradiation. *Nat. Commun.* 6:8567 doi: 10.1038/ncomms9567 (2015).

## Supplementary Material

Supplementary Information Supplementary Figures 1-5, Supplementary Tables 1-3, Supplementary Note 1 and Supplementary References

## Figures and Tables

**Table 1:** Intramolecular isotopic signatures and equilibrium fractionation factors.

	**δ*D***_**Benz**_	**δ*D***_**Aliph**_	**δ*D***_**Arom**_	***α***_**Benz-Arom**_	***α***_**Aliph-Arom**_
This study	1,192±173	463±113	81±128	2.03±0.29	1.35±0.19
Remusat *et al*.^9^	1,250±50	550±50	150±50	1.96±0.05	1.35±0.05
Robert *et al*.^24^				1.99±0.38	1.22±0.39

This table provides experimental isotopic signatures for benzilic (δ*D*_Benz_), aliphatic (δ*D*_Aliph_) and aromatic (δ*D*_Arom_) C–H functional groups determined from the irradiated samples. Derived equilibrium fractionation factors between these groups are also reported (*α*_Benz-Arom_ and *α*_Aliph-Arom_). Measurements on the Orgueil (CI) chondrite^9^ and results of the experimental exchange experiments^24^ are reproduced for comparison.
