# Supplementary material for: The deuterium/hydrogen distribution in chondritic organic matter attests to early ionizing irradiation
Source: Nat Commun. 2015 Oct 13;6:8567. doi: 10.1038/ncomms9567 (PMC4633821; doi:10.1038/ncomms9567)
Supplement: Supplementary Information — Supplementary Figures 1-5, Supplementary Tables 1-3, Supplementary Note 1 and Supplementary References [file ncomms9567-s1.pdf]

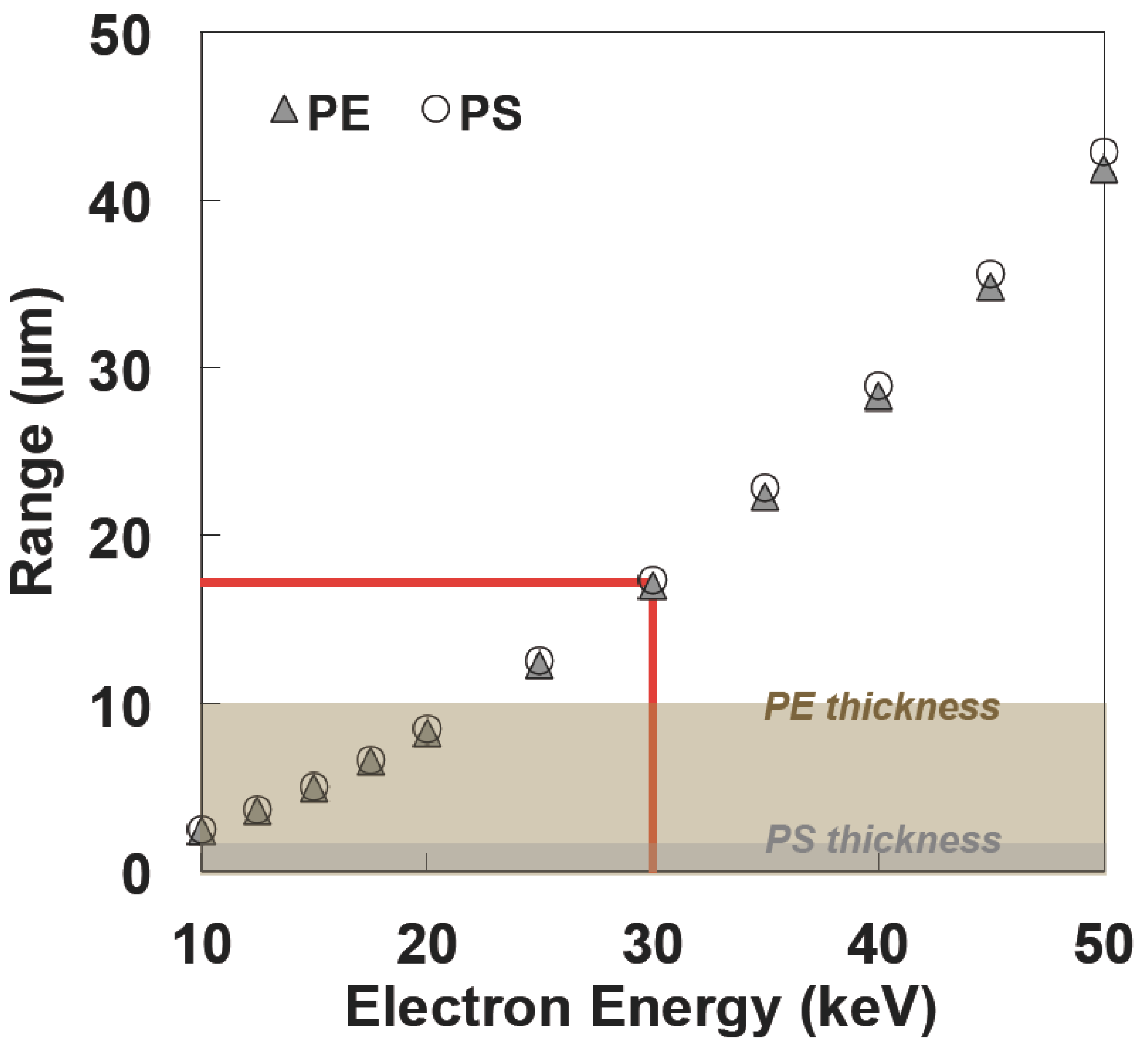

Supplementary Figure 1. Penetration depth of the electrons as a function of the incoming electron energy. Data for polystyrene (PS - circles) and polyethylene (PE - triangles). Electron ranges are taken from the ESTAR database.

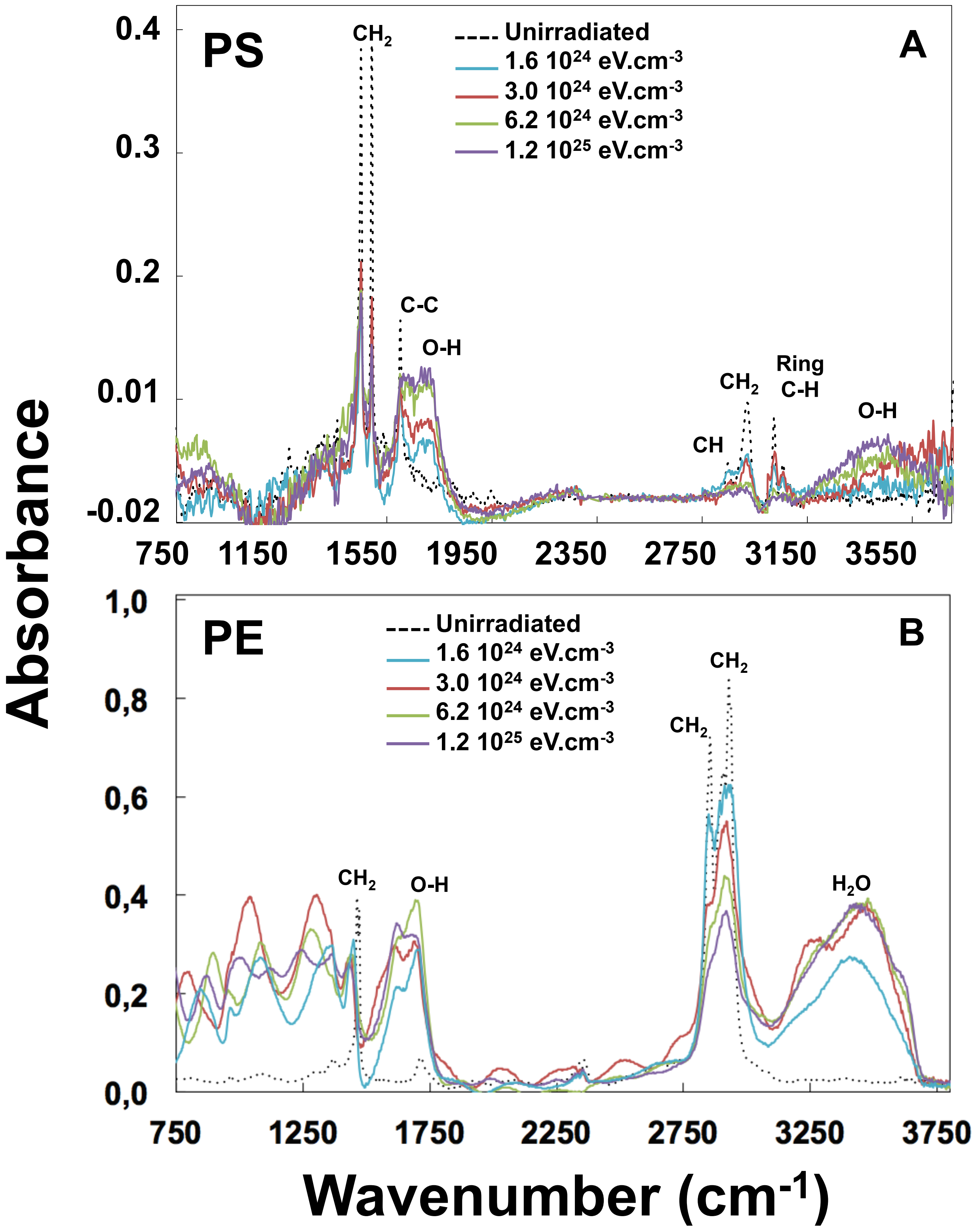

Supplementary Figure 2. Typical Fourier Transform Infrared absorbance spectra of the starting and the irradiated materials. Plots are for (a) polystyrene (PS) and (b) polyethylene (PE). The films were irradiated with 30 keV electrons at 300 K for different electron doses.

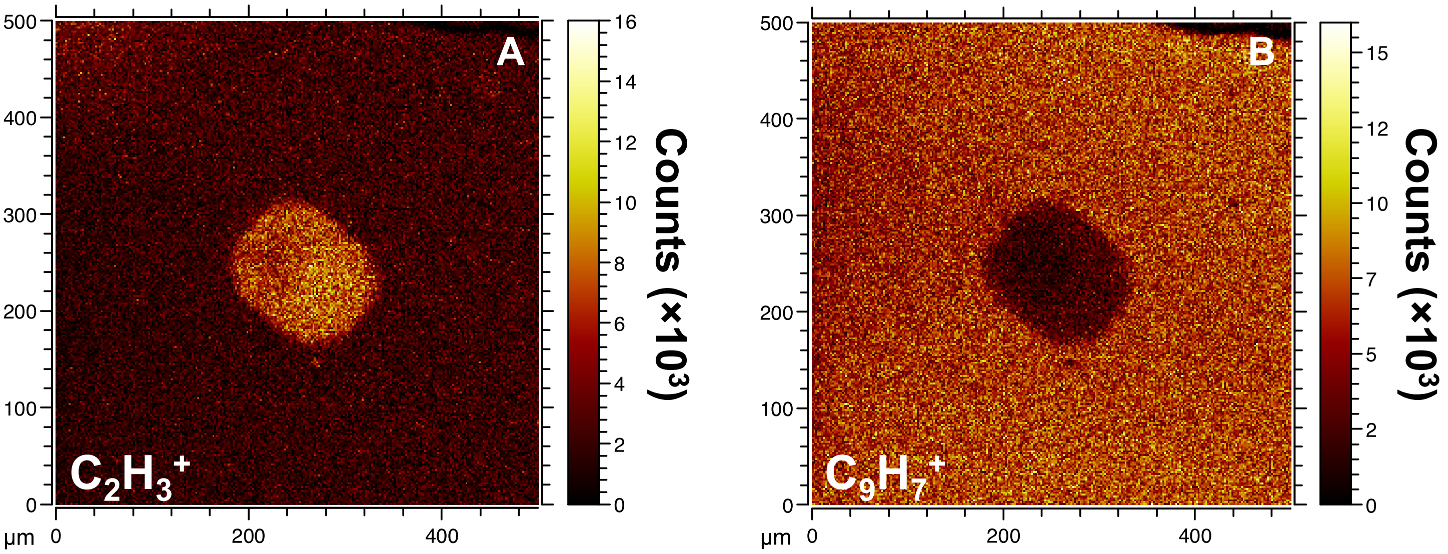

Supplementary Figure 3. Evolution of the carbon skeleton of polystyrene under irradiation imaged by ToF-SIMS. The polystyrene (PS) film was irradiated at 30 keV at 300K. The electron dose was 1.6 10^{24} eV.cm^{-3}. (a) The short fragments (here $C_2H_3^+$) are more abundant in the irradiated zone (centre spot) than in pristine zones. (b) Conversely, heavier fragments such as $C_9H_7^+$ are less abundant in the irradiated zone.

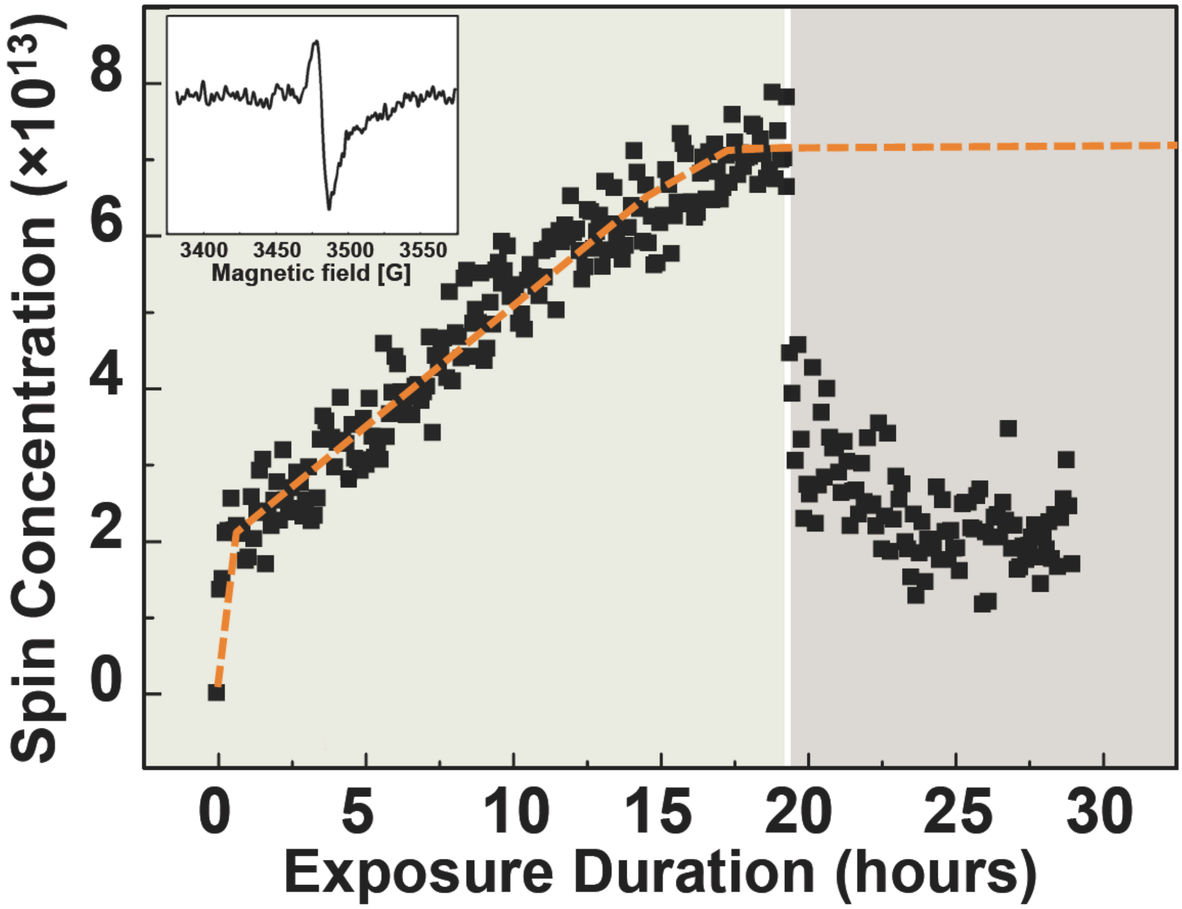

Supplementary Figure 4. Radical concentration measured by *in situ* EPR spectroscopy during UV irradiation. A clear one-line spectrum centred at g=2.004 with a linewidth of 10 G is observed as soon as the UV irradiation proceeds (inset). Such a spectrum is unambiguously attributed to a carbon-centred radical. The spin concentration increases during irradiation (light grey area), but a fast recombination of these radicals is observed when the UV source is turned off (darker grey area).

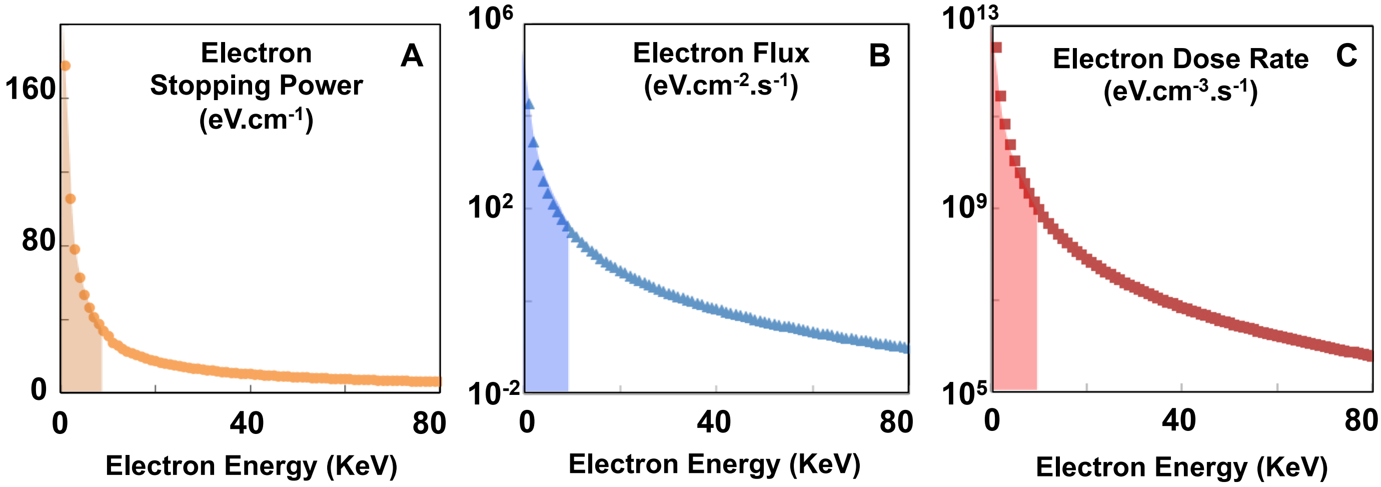

Supplementary Figure 5. Electron stopping power, flux and dose rate. (a) Electron stopping power (here for polyethylene, PE) as a function of the electron energy[1]. (b) Electron flux in the present-day solar flux[2]. (c) The corresponding electron dose rate was calculated from the integration of the stopping power and the electron flux in a low energy regime (colored zones).

Supplementary Table 1: Experimental conditions and results.

This table details the experimental conditions (energy of the incoming electron beam, run durations, electronic stopping powers of the three starting materials, electron fluences and doses). Isotopic compositions of hydrogen (δD), chemical ($H^+/C_2H_4^+$) information and dose constants are also reported.

Sample	Energy (keV)	Irradiation Duration (s)	Electronic stopping Power (MeV.cm^{-1})	Electron Fluence (eV.cm^{-2})	Electron Dose (eV.cm^{-3})	δD vs. SMOW (‰)	1-($H/C_2H_4^+$) (normalized)	Dose Constant (cm^3.eV^{-1})
		900		$1.8\ 10^{17}(\pm 3.10^{16})$	$2.0\ 10^{24}(\pm 3.10^{23})$	349 (±48)	0.48 (±0.20)	
**PE**	30	1800	11	$3.3\ 10^{17}(\pm 5.10^{16})$	$3.7\ 10^{24}(\pm 7.10^{23})$	392 (±145)	0.84 (±0.15)	$4.0\ 10^{-25}$
		3600		$7.0\ 10^{17}(\pm 1.10^{17})$	$7.7\ 10^{24}(\pm 1.10^{24})$	537 (±198)	1.00 (±0.12)	
		5400		$1.3\ 10^{18}(\pm 2.10^{17})$	$1.4\ 10^{25}(\pm 3.10^{24})$	488 (±84)	0.94 (±0.20)	
		900		$1.8\ 10^{17}(\pm 3.10^{16})$	$2.3\ 10^{24}(\pm 3.10^{23})$	244 (±60)	0.38 (±0.20)	
**PET**	30	1800	13	$3.4\ 10^{17}(\pm 5.10^{16})$	$4.4\ 10^{24}(\pm 7.10^{23})$	333 (±149)	0.77 (±0.10)	$4.0\ 10^{-25}$
		3600		$7.1\ 10^{17}(\pm 1.10^{17})$	$9.2\ 10^{24}(\pm 1.10^{24})$	287 (±84)	0.88 (±0.12)	
		5400		$1.3\ 10^{18}(\pm 2.10^{17})$	$1.7\ 10^{25}(\pm 3.10^{24})$	286 (±93)	1.00 (±0.18)	
		900		$1.7\ 10^{17}(\pm 3.10^{16})$	$1.6\ 10^{24}(\pm 3.10^{23})$	191 (±28)	0.50 (±0.04)	
**PS**	30	1800	9	$3.3\ 10^{17}(\pm 5.10^{16})$	$3.0\ 10^{24}(\pm 5.10^{23})$	247 (±19)	1.00 (±0.05)	$5.0\ 10^{-25}$
		3600		$6.9\ 10^{17}(\pm 1.10^{17})$	$6.2\ 10^{24}(\pm 1.10^{24})$	316 (±39)		
		5400		$1.3\ 10^{18}(\pm 2.10^{17})$	$1.2\ 10^{25}(\pm 2.10^{24})$	327 (±15)	0.92 (±0.16)	

Supplementary Table 2: Electron dose required to reach 90% of the plateau

Sample	Dose required to reach 90% of the isotopic plateau ($eV.cm^{-3}$)	Error (1σ)
**PE**	$6.0\ 10^{24}$	$1.0\ 10^{24}$
**PS**	$4.8\ 10^{24}$	$8.0\ 10^{23}$
**PET**	$5.8\ 10^{24}$	$1.0\ 10^{24}$

Supplementary Table 3: Determination of the timescale for a similar irradiation in the protoplanetary disk.

This table provides the average stopping power, integrated flux and associated electron dose rates for the three different samples based on current solar flux[2] for which electrons in the range 1-10 keV are predominant compared with those with higher energies. A typical exposure time in the protoplanetary disk (flux enhanced by 3 orders of magnitude compared with the present-day flux) is deduced from the integrated dose rate.

	Energy Range (keV)	Average stopping power ($MeV.cm^{-1}$)	Integrated flux ($e^{-}.cm^{-2}.s^{-1}$)	Integrated dose rate ($eV.cm^{3}.s^{1}$)	Time (years)
**PE**	1-10	48	13800	$1.5\ 10^{12}$	$1.5\ 10^{2}$
**PS**	1-10	50	13800	$1.5\ 10^{12}$	$1.5\ 10^{2}$
**PET**	1-10	67	13800	$2.0\ 10^{12}$	$1.1\ 10^{2}$

Supplementary Note 1: Effects of the UV irradiation

The UV irradiation also induces structural and isotopic modifications of the PET film. The *in situ* EPR measurements show that monoradicals are produced as soon as irradiation starts (Supplementary Figure 4). Their concentration increases linearly and then might level off after 15 hours (1.7 10^{24} eV.cm^{-3}), although no plateau can be resolved during this *in situ* experiment. At a comparable dose of 2.3 10^{24} eV.cm^{-3}, both electron[3] and photon irradiations of PET produce carbon-centred monoradicals of the same nature (a clear one-line spectrum centred at g=2.004 with a linewidth of 10 G). Interestingly, as soon as the UV source is turned off, a fast recombination of the defects occurs. Consequently, the amount of residual radicals in the PET film, as observed after a few minutes, is low (Supplementary Figure 4). Such an *in situ* EPR experiment cannot be carried out during electron irradiation. Nonetheless, this fast recombination seems at odds with the results from samples irradiated with electrons, for which a large amount of both monoradicals and diradicals were recovered several months after the electron irradiation. This may simply reflect the large difference between the typical penetration depths of the two types of particles. In contrast with electrons, which interact with the organic matter over more than 10 microns, UV photons at 329 nm are strongly absorbed within the first 100 nm. In other words, whereas these photons only interact with the surface of the sample, electrons induce bulk modification of the structure, and radicals formed in the bulk could be preserved over time.

Turning to the isotopic signatures, the δD values measured on samples irradiated with electrons and UV radiation are very similar for a given deposited energy. Typically, the δD is 224±50‰ for a UV dose of 3.1 10^{24} eV.cm^{-3} and is 278±12‰ for an electron dose of 2.3 10^{24} eV.cm^{-3}. A more systematic exploration of the UV irradiation process is still needed to better compare the effects of electron and UV radiation, but these results do not point to major differences. The only clear difference is the thickness of the materials affected by the irradiation (penetration depths of the incoming particles). The penetration depth is restricted to the first 100 nm for UV and exceeds a few microns for electrons accelerated at 30 keV.

Supplementary References:

[1] Berger, M. J., Coursey, J. S., Zucker, M. A., & Chang, J. *ESTAR, PSTAR, and ASTAR: Computer Programs for Calculating Stopping-Power and Range Tables for Electrons, Protons, and Helium Ions* (version 1.2.3). [Online] Available: http://physics.nist.gov/Star [*2012, 06 01*]. National Institute of Standards and Technology, Gaithersburg, MD (2005).

[2] Lin, R. P. *et al.* A three-dimensional plasma and energetic particle investigation for the WIND spacecraft. Space Sci. Rev., **71**, 125-153 (1995).

[3] Laurent, B. *et al.,* Isotopic and structural signature of experimentally irradiated organic matter. Geochim. Cosmochim. Acta., **142**, 522-534 (2014).
